# Mortality among patients with schizophrenia and vocational rehabilitation program services under Taiwan’s psychiatric care reform

**DOI:** 10.1186/s13033-016-0063-9

**Published:** 2016-04-12

**Authors:** Kan-Yuan Cheng, Shu-Yuan Chen, Chih-Yuan Lin

**Affiliations:** Department of Psychiatry, Taipei Veterans’ General Hospital Yuli Branch, No. 91, XinXing Rd., Yuli Township, 980 Hualien County Taiwan; Department of Public Health, Tzu Chi University, No. 701, Zhongyang Rd., Sec. 3, Hualien City, 970 Hualien County Taiwan

**Keywords:** Death rate, Supported employment, Non-vocational outcome, Community facility

## Abstract

**Background:**

Vocational rehabilitation programs are implemented to enhance the occupational functioning of long-stay patients with schizophrenia. Unemployment is associated with a higher risk of death. Schizophrenia patients who participate in vocational rehabilitation programs may have better health outcomes with participation in employment.

**Aim:**

To evaluate the relationship between mortality among schizophrenia patients and vocational rehabilitation program services under Taiwan’s psychiatric care reform.

**Methods:**

A total of 2457 long-stay schizophrenia patients were followed-up retrospectively from 1998 to 2008 at Taipei Veterans General Hospital Yuli Branch in Taiwan. We collected data on annual measurements of effectiveness and the human resources utilized in the vocational rehabilitation program. Pearson’s correlations between the above-collected data and the crude death rates for all patients were examined. We also assessed the association between participation in supported or sheltered employment and death.

**Results:**

Most of the patients were male (81.3 %). The mean ± SD age of the patients was 57.8 ± 17.0 years. The annual crude death rate averaged 5.3 %. Both the number of community workplaces and the total wages earned from sheltered and supported employment had significantly negative linear correlations with the crude death rate among all patients (both γ ≤ −0.64, p < 0.05). After controlling the confounding factors, participation in supported or sheltered employment was significantly associated with a lower risk of death (n = 2174, HR = 0.22, 95 % CI 0.16–0.29).

**Conclusions:**

Under psychiatric care reform, the vocational rehabilitation program was more effective and there was less patient mortality. Patients who had experienced sheltered or supported employment had a lower risk of death than those who had not.

## Background

Patients with schizophrenia have a higher mortality than the general population, due to both natural and unnatural causes [1, 2]. Unhealthy lifestyles [3], adverse effects of antipsychotics [4], physical comorbidities [5] and vulnerability to suicide [6] have been reported to increase the risk of death in patients with schizophrenia. In addition, schizophrenia patients have poorer occupational functioning and higher unemployment rates than the general population [7, 8]. Unemployment is also associated with higher mortality [9–11]. Unhealthy behavior, substance use, physical illness and suicide could contribute to the higher mortality risk of unemployed persons [12–14]. These factors are similar to the reported risk factors for death among schizophrenia patients. Therefore, unemployment might be related to the elevated risk of death among schizophrenia patients.

In the course of schizophrenia, unemployment can be a result of illness prognoses or initial social underachievement. Under psychiatric care reform, long-stay patients with schizophrenia can participate in vocational rehabilitation programs to improve their occupational and social functioning [15]. However, several studies have found that mortality gaps still exist between psychiatric patients and the general population in post-deinstitutionalization periods [16–19]. In presenting the service changes that accompany psychiatric care reform, studies usually describe the national policy on establishing community facilities or reducing the number of beds in psychiatric institutions [6, 19, 20]. Because the actual numbers of psychiatric rehabilitation program services are seldom discussed, the relationship between participation in psychiatric rehabilitation programs and risk of death among schizophrenia patients is still unclear.

Since 1995, the National Health Insurance program in Taiwan has paid for the services of community rehabilitation centres and recovery homes that promote community-based mental health care. In 2014, a total of 3458 beds in 73 community rehabilitation centers and 4961 beds in 117 recovery homes (half-way houses) had already been established in Taiwan. Taipei Veterans General Hospital Yuli Branch (VGH-YL) in eastern Taiwan is a psychiatric teaching hospital that accommodates patients with severe mental disability from all over Taiwan. The hospital has a capacity of 120 beds in acute wards, 731 beds in chronic wards, 220 beds in a day ward, 1899 beds in nursing-home wards, 280 beds in a recovery home and 220 beds in community rehabilitation centers for psychiatric care. Over the past 20 years, VGH-YL has reformed psychiatric care for patients with severe mental disability [21]. In 1998, a vocational rehabilitation program was set up in inpatient settings and community facilities for long-stay patients with severe psychiatric disability [21, 22]. Occupational therapy, industrial therapy and prevocational training were mainly provided in the acute, chronic and nursing-home wards. After leaving the inpatient setting, patients could participate in sheltered employment and supported employment under the services provided by the recovery home and two community rehabilitation centers in Yuli town [21, 22].

In addition to vocational outcomes, vocational rehabilitation programs can also enhance non-vocational outcomes, such as the health, quality of life and social functioning of patients with schizophrenia [23–25]. Vocational rehabilitation can improve the occupational functioning of schizophrenia patients and have positive effects on their health. Better health might then reduce the risk of death. Some studies have shown improvements in mortality among patients with severe mental illness after psychiatric care reform [26–28]. An earlier study also revealed decreased mortality gaps between long-stay patients with schizophrenia and the general population under psychiatric care reform in VGH-YL [29]. Based on these findings, this study was designed to evaluate the relationship between mortality among long-stay patients with schizophrenia and vocational rehabilitation services in the case of VGH-YL. The risk of death among patients who had participated in sheltered or supported employment was also investigated.

## Methods

### Study sample

Using the digital inpatient registration database at VGH-YL in Taiwan, a total of 2457 patients who had admission diagnoses of schizophrenia and who had been hospitalized for at least 1 year before Dec 31, 1997 were recruited [29]. As there was no age limit for the vocational rehabilitation program, and some elderly patients still participated in sheltered employment in farming, we did not exclude patients who were over 65 years of age. All eligible patients were retrospectively followed-up from Jan 1, 1998 to Dec 31, 2008. We linked to the National Death Certification System in Taiwan using the identification numbers of all recruited patients to identify those who had died in an inpatient setting or community facility. Patients who had declined the care of VGH-YL were viewed as withdrawals, except for patients who had interrupted hospitalization for fewer than 31 days or died after transferal for advanced medical treatment. All patients who died after transferal due to medical conditions were viewed as decedents during follow-up [29]. We calculated the crude death rate of the study population for each year.

### Data collection

Data on the annual numbers of patients who had experienced supported employment, their workplace in Yuli Township, and the total wages earned from their sheltered and supported employment as entered in the files of the Rehabilitation Foundation of VGH-YL were recorded. The above data were collected to analyze the effectiveness of the vocational rehabilitation program. Each December, we collected data on the numbers of job coaches and occupational therapists who were involved in the vocational rehabilitation program from the manpower files of the Department of Rehabilitation in VGH-YL.

Demographic, psychiatric history and physical comorbidity data for each patient were also retrieved from the digital inpatient registration database. The welfare status of the patient was based on data from social security resources in Taiwan. Patients who had a VGH-YL address as their census registration may not have had a family, or their family did not want the patient to use the address in the census registration. Data on all physical illnesses diagnosed in inpatient and outpatient departments were collected for the whole follow-up period. Classification of physical comorbidity was based on the major International Classification of Diseases, 9th Revision codes according to the physiopathology, affected organs or physiological systems. As the recovery home in Yuli town offers sheltered or supported employment services, living in a recovery home for at least 6 months was considered as stable participation in sheltered or supported employment. The decedents and survivors at the end of follow-up were both included to evaluate the factors associated with risk of death using univariate and multivariate analyses.

### Statistical methods

In univariate analysis, the Chi square test was used for categorical variables, and the independent *t* test and Mann–Whitney U test were used for continuous variables with and without a normal distribution. The Cox proportional regression model was used to estimate the risk of death in multivariate analysis. All statistics were calculated using the Statistical Package for the Social Sciences for Windows (version 15.0; SPSS Inc., Chicago, Illinois), and all tests were two-tailed. The significance level was set at 0.05.

### Ethics statement

Ethical approval of the study was provided by the Institutional Review Board of Taipei Veterans General Hospital Yuli Branch (99-08-02A). We protected the privacy of study subjects by removing information that could be used for identification.

## Results

### Characteristics of the subjects

The mean ± SD age of the study subjects was 57.8 ± 17.0 years. A total of 1998 patients were male (81.3 %) and 2124 patients were unmarried (86.4 %). Two out of three patients were veterans (n = 1652, 67.2 %). Nearly half of all patients had VGH-YL as their address in their census registration (n = 1183, 48.1 %). Only 102 patients (4.2 %) had a psychiatric diagnosis other than schizophrenia. Two out of three patients had physical comorbidity (n = 1684, 68.5 %). In all, 335 patients (13.6 %) had participated in sheltered or supported employment. In the follow-up period, 993 (40.4 %) died, and 280 (11.4 %) withdrew [29].

### The crude death rate and the service amounts of the vocational rehabilitation program

**Fig. 1:**
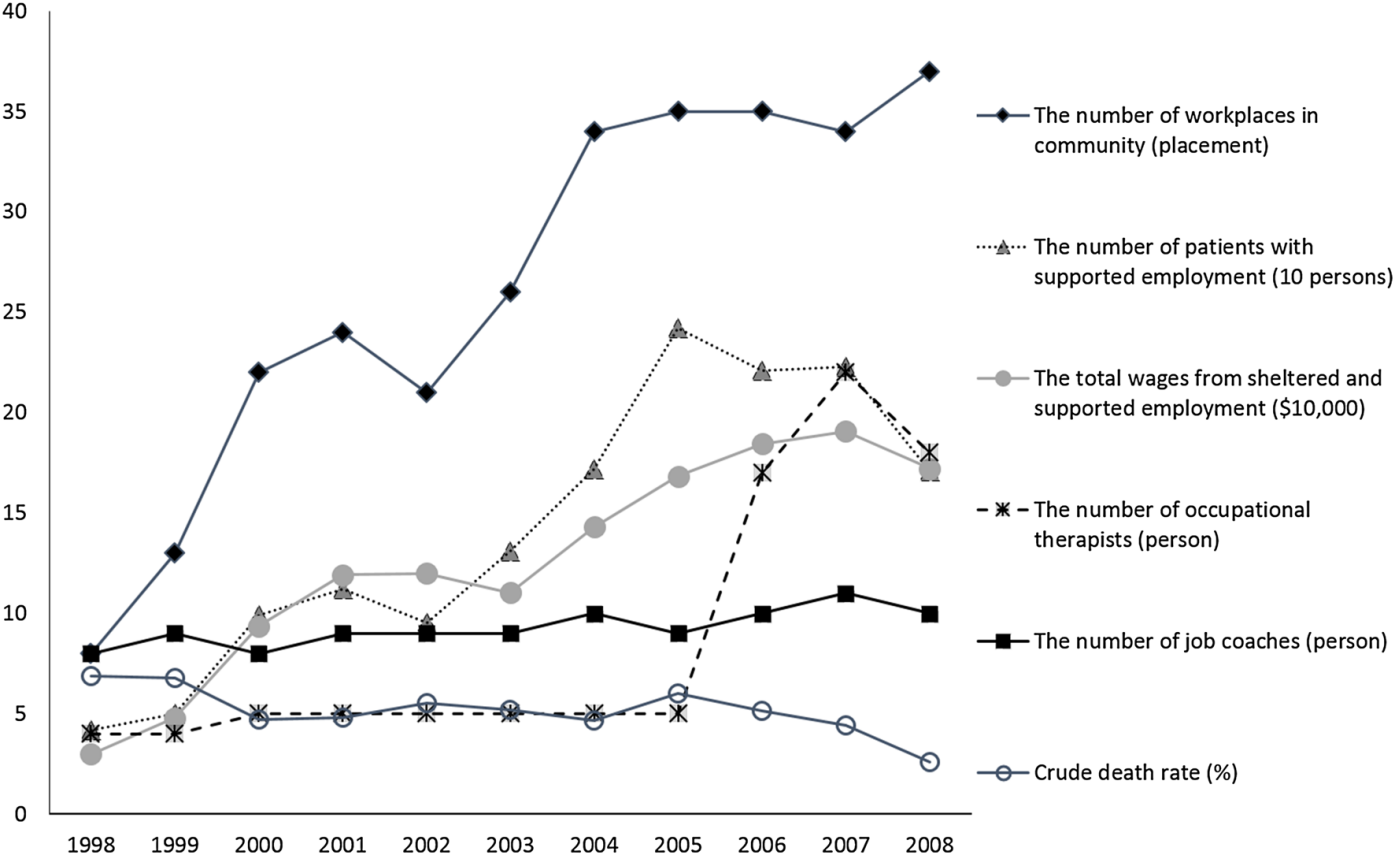
Crude death rates of all patients and measurements of effectiveness of and human resource utilization in the vocational rehabilitation program

The mean of the crude death rate for all study subjects was 5.3 % per year between 1998 and 2008. The highest was 6.9 % in 1998 and the lowest was 2.6 % in 2008 (Fig. 1). At the end of follow-up, 22.9 % of the surviving patients lived in a community facility (n = 335). Throughout the follow-up period, the average rate of supported employment was 8.4 % per year. The highest was 17.6 % in 2007 and the lowest was 1.8 % in 1998. Both the number of patients with supported employment and the total wages earned for sheltered and supported employment grew rapidly before 2005, but diminished in 2008 (Fig. 1). However, the number of workplaces in Yuli town continued to grow in 2008 (Fig. 1). With regard to manpower in the vocational rehabilitation program, the number of occupational therapists clearly increased in 2006 and 2007, but the number of job coaches increased only slightly in the same period (Fig. 1).

The measurement of effectiveness and the amounts of human resources utilized had significantly positive linear correlations with each other (Table 1). The number of workplaces in Yuli town (r = −0.64, p < 0.05) and the total wages from sheltered and supported employment (r = −0.70, p < 0.05) had significantly negative linear correlations with the crude death rate of all the study subjects.

**Table 1:** The relationship between mortality among the 2457 patients with schizophrenia and vocational rehabilitation program services in Taipei Veterans’ general hospital Yuli branch in Taiwan from 1998 to 2008

Pearson’s correlation	Effectiveness			Human resource^a^	
	Supported employment^b^	Workplaces in community	Total wages^c^	Occupational therapists	Job coaches
Annular crude death rate	−0.44	−0.70^d^	−0.64^d^	−0.60	−0.52
Supported employment^b^		0.93^e^	0.94^e^	0.62^d^	0.71^d^
Workplaces in community			0.96^e^	0.62^d^	0.74^e^
Total wages^c^				0.72^d^	0.79^e^
Occupational therapists					0.80^e^

^a^The numbers of specialists who had served in vocational rehabilitation program

^b^The number of those with participations in supported employment

^c^Combined the incomes of sheltered and supported employment

^d^p < 0.05

^e^p < 0.01

### Comparisons between the patients with sheltered or supported employment and those without

The patients with sheltered or supported employment were significantly younger than those without (n = 335, 52.6 ± 17.5 vs. n = 2122, 58.7 ± 16.7, t = 5.9, p < 0.01), with a shorter duration of hospitalization (n = 335, 5.8 ± 8.8 vs. n = 2122, 8.7 ± 10.9, t = 5.6, p < 0.01). Higher percentages of male subjects and those of an unmarried status were noted in the patients with sheltered or supported employment than in those without (n = 317, 94.6 % vs. n = 1681, 79.2 %, X^2^ = 45.2, p < 0.01; n = 321, 95.8 % vs. n = 1803, 85.0 %, X^2^ = 30.8, p < 0.01, respectively). The patients with sheltered or supported employment had significantly lower proportions of psychiatric or physical comorbidity (n = 6, 1.8 % vs. n = 96, 4.5 %, X^2^ = 5.4, p = 0.02; n = 213, 63.6 % vs. n = 1471, 69.3 %, X^2^ = 4.4, p = 0.04, respectively). The patients with sheltered or supported employment had a clearly lower death rate than the patients without (n = 50, 14.9 % vs. n = 943, 44.4 %, X^2^ = 103.4, p < 0.01).

### Comparison of survivors and decedents

The decedents were older and included significantly higher percentages of males, those who were married, veterans and those with a census registration address of the hospital as compared with the survivors (all p < 0.001) (Table 2). The deceased patients also had significantly more physical comorbidity for nearly all systemic diseases than the survivors (Table 2). Compared with the survivors, the decedents clearly had longer durations of hospitalization and mental illness, but lower percentages of those who experienced the whole rehabilitation program (all p < 0.001) (Table 2).

**Table 2:** Characteristics of the 1184 surviving and 993 deceased patients with schizophrenia under care at Taipei Veterans’ General Hospital Yuli Branch in Taiwan from 1998 to 2008

Characteristics	Decedents		Survivors		P
	N	%	N	%	
Demography					
Age, Mean ± SD^a^	69.22 ± 10.46		48.79 ± 15.38		<0.001
Male	934	94.1	844	71.3	<0.001
Marital status					
Unmarried	833	83.9	1047	88.4	<0.001
Married	126	12.7	51	4.3	
Widow	7	0.7	64	5.4	
Divorced	27	2.7	22	1.9	
Social welfare^b^					
Veterans	912	91.8	573	48.4	<0.001
Relatives of veterans	62	6.2	438	37.0	
Low-income	11	1.1	103	8.7	
General	8	0.8	70	5.9	
Census registration					
Hospital^c^	749	75.4	319	26.9	<0.001
Non-Hospital	244	24.6	865	73.1	
Psychiatric history					
Hospitalization duration in the initial follow-up (years), Median, 25–75 percentile^a^	2.8, 2.0–17.9		2.8, 2.0–6.4		<0.001
Duration of mental illness, Median, 25–75 percentile^a^	9.9, 5.7–22.3		13.9, 13.0–17.4		<0.001
Other combined psychiatric diagnoses	29	2.9	51	4.3	0.087
Participation in sheltered or supported employment	50	5.0	276	23.3	<0.001
Physical comorbidity (ICD9)	804	81.0	697	58.9	<0.001
Diseases of the respiratory system (460–519)	455	45.8	220	18.6	<0.001
Diseases of the digestive system (520–579)	328	33.0	297	25.1	<0.001
Symptoms, signs, and ill-defined conditions (780–799)	223	22.5	200	16.9	0.001
Diseases of the genitourinary system (580–629)	215	21.7	115	9.7	<0.001
Infectious and parasitic disease (001-139)	194	19.5	101	8.5	<0.001
Diseases of the circulatory system (390–459)	178	17.8	104	8.8	<0.001
Diseases of the skin and subcutaneous tissue (680–709)	178	17.9	178	15.0	0.069
Endocrine, nutritional and metabolic diseases, and immunity disorders (240–279)	107	10.8	92	7.8	0.015
Neoplasms (140–239)	78	7.9	28	2.4	<0.001
Diseases of the nervous and sensory organs (320–389)	69	7.0	44	3.7	0.001
Diseases of the musculoskeletal system and connective tissue (710–739)	57	5.7	62	5.2	0.607
Diseases of the blood and blood-forming organs (280–289)	39	3.9	25	2.1	0.012

*ICD 9* International Classification of Diseases, 9th edition

^a^Mann-Whitney U test

^b^The classifications are based on social welfare resources in Taiwan

^c^Those with no family or who refused to use the same registration census as their family in Taiwan

The male patients had a higher risk of death than the female patients (hazard ratio (HR) = 2.18, 95 % CI 1.49–3.20). Patients had a 6 % higher risk of death with each one-year increase in age (HR = 1.06, 95 % CI 1.05–1.07). Compared with the unmarried patients, the married and widowed patients had a higher risk of death, with HRs of 2.22 (95 % CI 1.83–2.71) and 1.72 (95 % CI 1.10–2.70), respectively. Patients with a census registration address of the hospital had a significantly higher risk of death than the others (HR = 1.25, 95 % CI 1.03–1.52, p = 0.02), and patients who had suffered from neoplasms had a higher risk of death, with an HR of 1.40 (95 % CI 1.06–1.86, p = 0.02), than those who had not. The patients who had participated in sheltered or supported employment had a lower risk of death than those who had not (HR = 0.21, 95 % CI 0.16–0.28, p < 0.001) (Table 3).

**Table 3:** Analysis of death risk using the Cox proportion regression model

Variable	Hazard ratio	95 % CI	P
Sex (male = 1, female = 0)	2.18	1.49–3.20	<0.01
Age (per 1 year)	1.06	1.05–1.07	<0.001
Marital status (married = 1, unmarried = 0)	2.22	1.83–2.71	<0.001
Marital status (widowed = 1, unmarried = 0)	1.72	1.10–2.70	0.04
Census registration (hospital^a^ = 1, non-hospital = 0)	1.25	1.03–1.52	0.02
Comorbidity of neoplasms (yes = 1, no = 0)	1.40	1.06–1.86	0.02
Participation in sheltered or supported employment (yes = 1, no = 0)	0.21	0.16–0.28	<0.001

*CI* confidence interval

^a^Those with no family or who refused to use the same registration census as their family in Taiwan

## Discussion

Both the number of patients with supported employment and the total wages earned from sheltered and supported employment dropped in 2008 (Fig. 1), when Taiwan went into an economic recession. However, the number of workplaces in Yuli town still increased that year (Fig. 1). The explanation might be that fewer employees with mental illness were hired in a single workplace due to the economic recession, but the number of workplaces in the community still grew because more employers welcomed workers with mental illness. A greater number of workplaces might increase the opportunities of persons with psychiatric disability to find a match in the labor market. The number of workplaces actually had a significantly negative linear relationship with the crude death rate of all patients (Table 1). Therefore, acceptance of those patients by more employers could facilitate social integration, which might enhance their mental health.

Total wages also had a significantly negative linear relationship with the crude death rate of all patients, but the amounts of human resources and number of patients with supported employment did not (Table 1). Total wages consists of the combined effectiveness of total hours worked and hourly earnings on the outcomes of the vocational rehabilitation programs [30]. Measuring total wages to represent overall effectiveness might be useful in evaluating the health care quality of vocational rehabilitation programs.

After adjusting the confounding factors, the male patients had a higher risk of death than the female patients. A study in Finland reported similar findings, in that 164 male long-stay schizophrenia patients had a higher death rate than 88 female patients (27.1 % vs. 18.9 %) between 1992 and 2000 [16]. The death rate among 7163 male inpatients with schizophrenia (17.4 %) was also much higher than that of 2876 female patients (7.4 %) in a 6-year follow-up study in Taiwan [31]. In our study, the risk of death of schizophrenia patients increased by 6 % for each additional year of age. This was supported by the results of an earlier American study, which revealed that the risk of death for elderly patients with mental illness increased by 4 % for each 1 year of age (RR = 1.04, 95 % CI 1.02–1.05) [32].

Married and widowed patients both had a higher risk of death than unmarried patients in our study. A study in Italy also reported that married (62.9 %) and widowed psychiatric inpatients (60.7 %) had a higher proportion of deaths than unmarried patients (47.7 %) [33]. The death rate of married (37.5 %) and widowed long-stay psychiatric patients (100 %) in an earlier study in Finland was also higher than that of unmarried patients (20.9 %) [16]. In our study, the factor of a census registration address in the hospital was correlated with an increased risk of death of the patients. This might be explained by the fact that patients who had a census registration address in the hospital had little or no support from their relatives to participate in the vocational rehabilitation program. Therefore, the role of family support in the quality of health care under the reform of psychiatric care should not be ignored.

Neoplasm was the only physical comorbidity that was identified a risk factor for death in these patients. This finding was compatible with the results of a previous study in Taiwan, which found that patients with schizophrenia had a lower cancer incidence, but a higher risk of death than the controls after adjusting other factors in a nine-year follow-up period [34]. Unlike other physical illnesses, adequate treatment for cancer, which requires accurate staging and comprehensive planning, is only offered in medical centers in Taiwan. Access to health care for cancer treatment could be related to the elevated risk of death in patients with schizophrenia who suffer from neoplasm.

Multivariate analysis revealed that participation in sheltered or supported employment decreased the risk of death among patients with schizophrenia. This finding was supported by the results of a previous study in rural Ireland. In a 20-year follow-up period, 297 psychiatric patients who were involved in a rehabilitation program were found to have a low death rate after relocation [35]. A nationwide registration study of a Nordic welfare state model found a diminishment in the life expectancy gap for psychiatric patients from 1987 to 2006, which also demonstrated the role of psychiatric rehabilitation services in health care quality after the reform of psychiatric care [26].

Schizophrenia patients who participate in supported or sheltered employment might have a better financial ability [36] to deal with health problems than patients living in inpatient settings. Furthermore, patients who work in the community are better able to maintain their mental and physical wellbeing [23, 37], so as to find a better job in a competitive environment. With employment, patients with schizophrenia can have pride, self-esteem and effective strategies to cope with psychotic symptoms [36], which can reduce the risk of suicide. Also, patients with supported employment can have a better health status [37], as a result of regular physical exercise in traveling from the community facility to the workplace by walking or riding a bicycle, as with our study subjects. In addition, schizophrenia patients in Taiwan have an opportunity to decrease their smoking when they work in the community, because smoking is forbidden in public areas under Taiwan’s Tobacco Hazards Prevention Act. Steady employment is also related to a lower risk of substance use disorders among these patients [23]. Finally, patients with supported or sheltered employment also have a lower risk of coming into contact with infectious diseases or pneumonia than patients who stay in an inpatient setting.

Of a total of 280 withdrawals, 156 patients were relocated to other long-stay facilities, 107 returned home with or without referral to the outpatient department, 14 discharged themselves against medical advice, and six left without leaving a message [29]. Most of the withdrawals left VGH-YL under their own will or through arrangement by their family. Those that withdrew were significantly younger (n = 280, 55.6 ± 18.0 vs. n = 2177, 58.1 ± 16.8, t = 2.2, p = 0.03), but had a higher death rate than the patients who had persistently received care at VGH-YL from 1998 to 2008 (n = 150, 53.6 % vs. n = 993, 45.6 %, X^2^ = 6.0, p = 0.01). In the withdrawals, the patients who returned home had a clearly lower death rate than those who were relocated to other long-stay facilities (n = 16, 15.4 % vs. n = 121, 77.6 %, X^2^ = 94.3, p < 0.01). The death rate of the withdrawals who returned home was similar to that of the patients with sheltered or supported employment (n = 50, 14.9 %). We suppose that the withdrawals who returned home might have had more opportunities to receive other rehabilitation services in Taiwan and consequently better outcomes in terms of mortality than those who lived in other long-stay facilities.

In this study, the subgroup of patients of a younger age, the male gender, an unmarried status, shorter hospitalization duration and no psychiatric or physical comorbidity were more likely to participate in sheltered or supported employment. Following this study, further evaluations should be performed to clarify the reasons why the other subgroups declined to participate in the vocational rehabilitation program, except for elderly patients. Aged patients with schizophrenia might need interventions other than vocational services to improve their health status.

This study might be the first to evaluate the relationship between mortality among patients with schizophrenia and rehabilitation program services under psychiatric care reform in a country without deinstitutionalization. The relatively large number of cases and complete collection of both group and individual data are this study’s strengths. Furthermore, the measurement of effectiveness and human resource utilization in the vocational rehabilitation program could directly demonstrate the service amounts of reformed psychiatric care. However, the lack of information on lifestyle and exposure to psychiatric medications in the patients’ individual data was a study limitation. Another limitation was that the study population was limited to a middle-aged group with severe mental disability and long-term hospitalization.

## Conclusions

The better mortality outcome of the long-stay patients with schizophrenia under psychiatric care in this study could be associated with participation in the vocational rehabilitation program. Group data analysis showed a significant negative linear relationship between the crude death rate of these patients and the effectiveness of the vocational rehabilitation program. For the individuals, participation in supported or sheltered employment was related to a lower risk of death. The improvement in overall health care quality with involvement in the vocational rehabilitation program was a positive long-term outcome of psychiatric care reform for these patients. In light of our findings, the relationship between vocational rehabilitation programs and mental or physical health needs further evaluation to determine the mechanism that protects against premature death among long-stay schizophrenia patients.
